# Supplementary material for: First Patient-to-Patient Intrahospital Transmission of Clade I Candida auris in France Revealed after a Two-Month Incubation Period
Source: Microbiol Spectr. 2022 Sep 12;10(5):e01833-22. doi: 10.1128/spectrum.01833-22 (PMC9604096; doi:10.1128/spectrum.01833-22)
Supplement: Supplemental file 1 — Supplemental material. Download spectrum.01833-22-s0001.pdf, PDF file, 0.9 MB [file spectrum.01833-22-s0001.pdf]

Conduite à tenir devant un cas de colonisation ou infection à *Candida auris*

Version 1 : 10/03/2022

RAPPEL

Candida auris est une levure responsable d'infections potentiellement graves, le plus souvent associées aux soins, et d'épidémies hospitalières difficiles à contrôler en raison d'une persistance inhabituelle dans l'environnement. La pression exercée par l'utilisation des antifongiques est considérée comme un facteur important de son émergence (résistance au fluconazole > 90%).
Réservoir : Persistance plusieurs mois dans l'environnement et chez les personnes colonisées ou infectées. Ne fait pas partie de la flore humaine commensale classique. Il est acquis en milieu de soins.
Mode de transmission : contact direct entre personnes, via les mains des personnels, ou contact indirect via l'environnement et le matériel de soins contaminé.
La prévention de la transmission repose sur la désinfection rigoureuse des mains par friction hydro-alcoolique (FHA) avant et après chaque contact avec le patient et son environnement et sur le bionettoyage, et la désinfection de l'environnement du patient et du matériel partagé **avec un détergent-désinfectant spécifiquement actif sur *C. auris***.

DEPISTAGES

- **Laboratoire de microbiologie/mycologie** :
  - Vérifier que le laboratoire de l'hôpital est en capacité d'identifier rapidement les souches suspectes (la base de données MALDI-TOF inclut *C. auris*).
  - Vérifier avec le laboratoire les conditions de prélèvement (type d'écouvillon, milieu de transport…)
  - Prévenir le laboratoire avant l'envoi d'un prélèvement chez un patient suspect.
  - Mettre au point une alerte immédiate (électronique ou autre) de l'EOH par le laboratoire si identification d'un cas.
  - Selon les situations :
    - Screening à l'entrée : dépistages par culture (utilisation de milieux chromogènes)
    - Si découverte fortuite ou contexte épidémique, envisager la détection par qPCR spécifique pour dépister les contacts, (disponible au laboratoire de Saint Louis, réponse <48h après réception).
- Sites à dépister :
  - Screening à l'entrée : **creux axillaires et plis inguinaux avec un seul écouvillon** en commençant par les aisselles. En fonction du contexte clinique, d'autres sites peuvent être prélevés : nez, gorge, plaies, rectum, urines, sécrétions endotrachéales hémocultures si fièvre…
  - Dépistage des contacts : 1 écouvillon pour les creux axillaires et les plis inguinaux **ET** 1 écouvillon nasal
- **Dépistage ciblé** recommandé chez tout patient :
  - Rapatrié à partir d'une réanimation d'un pays étranger
  - Hospitalisé dans les 12 mois précédents dans une zone ou un établissement où une épidémie à *C. auris* est en cours. En février 2022, pays concernés : Moyen orient (Dubaï, Emirats arabes unis, Iran), Italie, USA, sous-continent indien, Afrique Australe, extrême Orient (Japon, Corée), Colombie/Venezuela. **Attention liste évolutive**.
  - Antérieurement colonisé ou infecté par *C. auris*
  - Contact d'un cas (selon situations décrites ci-dessous).

DEFINITIONS PATIENT CONTACT ET PATIENT SUSPECT

- **Contact à risque faible :** patient ayant séjourné dans la même unité de soins qu'un cas (infecté ou colonisé) alors que les mesures de prévention de la diffusion sont mises en œuvre.
- **Contact à risque intermédiaire :** patient ayant séjourné dans la même unité de soins qu'un cas sans que les mesures de prévention de la diffusion soient mises en œuvre (découverte fortuite).
- **Contact à risque élevé :** patient contact dans un contexte épidémique (au moins un cas secondaire a été identifié).

MESURES CONCERNANT LES PATIENTS PORTEURS ET A RISQUE DE PORTAGE, A APPLIQUER DANS TOUTES LES SITUATIONS

- **Précautions complémentaires contact en chambre individuelle** (FHA, protection de la tenue si contact direct avec le patient, gants si contacts avec les liquides biologiques…)
- **Bionettoyage biquotidien** de la chambre du patient, en insistant sur l'environnement proche du patient, incluant le matériel partagé, à l'aide d'un produit actif sur *C. auris* (Par ex. : Anios Oxy'floor®, Incidin Oxyfoam®, hypochlorite de sodium à 0.1% de chlore actif).
- A la sortie du patient, réaliser un bionettoyage rigoureux (voire double sur avis de l'EOH) avec un produit actif sur *C. auris* (insister sur le lit, le **matelas**, l'adaptable, le matériel partagé…).

- Si intervention chirurgicale, accès au plateau technique interventionnel ou imagerie : circuit du patient similaire à celui des patients colonisés à BHRe (planification en fin de programme, etc…) ET utilisation d'un détergent-désinfectant spécifique pour le bionettoyage après le passage du patient.

Matériels de soins et équipement médical :

- Utiliser du matériel médical à usage unique ou dédier le matériel dans la chambre du patient
- Le matériel qui ne peut être dédié doit être désinfecté à l'aide d'un produit actif sur *C. auris* entre deux patients
- Limiter la quantité de matériel de soins dans la chambre au strict nécessaire

Gestion des déchets, linge et vaisselle

Selon les procédures habituelles de l'établissement

SITUATION 1 : DECOUVERTE D'UN PATIENT PORTEUR POSITIF A L'ADMISSION

Patient porteur :

- A hospitaliser dans une chambre individuelle,
- Appliquer les précautions complémentaires contact (PCC) et les maintenir tout au long de l'hospitalisation
- Tracer le portage dans ORBIS (« alerte infectiovigilance »)
- Dédier du personnel paramédical ou, à défaut, appliquer la marche en avant

Patients contacts :

Réaliser un dépistage hebdomadaire des creux axillaires et des plis inguinaux (avec le même écouvillon) + un dépistage nasal (avec un 2e écouvillon) tant que le patient porteur est présent, par culture et/ou qPCR spécifique.
Aucune PCC n'est recommandée
Pas de dépistage ni de mesure particulière lors des futures admissions, pas de traçabilité informatique nécessaire
Transferts non restreints, secteur d'aval informé de la situation à risque faible de transmission, précautions standard.

SITUATION 2 : PRISE EN CHARGE D'UN PATIENT AUX ANTECEDENTS DE PORTAGE DE *C. AURIS*

- Appliquer les PCC et les maintenir tout au long de l'hospitalisation
- Réaliser un dépistage lors de la réadmission
  - Si résultat **négatif** :
    - Maintenir les PCC tout au long de l'hospitalisation
    - Dépister le cas 1x/semaine tout au long de l'hospitalisation
    - Pas de dépistage des autres patients de l'unité
  - Si résultat positif : cf. situation 1

SITUATION 3 : DECOUVERTE FORTUITE D'UN PATIENT PORTEUR DE C. AURIS EN COURS D'HOSPITALISATION

Dès le jour du résultat :

- Hospitaliser le patient dans une chambre individuelle
- Appliquer les PCC et les maintenir tout au long de l'hospitalisation
- Dédier du personnel paramédical ou, à défaut, respecter la marche en avant
- Tracer le portage dans ORBIS (« infectiovigilance »)
- Analyser les circonstances d'acquisition, chercher une éventuelle exposition à l'étranger
- Arrêt des transferts des patients de l'unité (porteur et contacts) en attendant le résultat des premiers dépistages
- Dépister tous les patients contacts présents (1 écouvillon creux axillaires/plis inguinaux + 1 écouvillon nasal) par culture et/ou qPCR spécifique sur écouvillons (contacter le laboratoire en amont).

Dans les jours suivants :

- Identifier au plus vite les patients contacts, en incluant ceux dont le contact remonte jusqu'à 4 semaines précédant le diagnostic et ceux déjà transférés dans d'autres services ou établissements
- Placer les contacts en PCC et les dépister 3 fois à une semaine d'intervalle,
- Si **aucun cas secondaire** n'est identifié lors des premiers dépistages :
  - Poursuivre les dépistages hebdomadaires des contacts tant que le porteur est présent dans l'unité

- Après la sortie d'hospitalisation du patient porteur, réaliser au moins 3 dépistages post-exposition des patients présents dans l'unité ; si aucun cas secondaire n'a été identifié, les dépistages sont arrêtés
- Autoriser les transferts. En cas de transfert d'un contact, PCC dans l'unité d'aval jusqu'à 3 dépistages négatifs post exposition (se mettre d'accord en amont pour une éventuelle analyse par le laboratoire de l'hôpital source).
- Pas de traçabilité informatique nécessaire pour les contacts

- Si **identification de cas secondaire(s)** : appliquer les mesures de contrôle d'une épidémie

SITUATION 4 : EPIDEMIE (AU MOINS UN CAS SECONDAIRE)

- Arrêter les transferts dans l'attente de la maîtrise de la situation
- Regrouper les cas, contacts et patients indemnes en 3 secteurs distincts, idéalement avec des équipes soignantes dédiées. Si marche en avant, éviter que les personnels prennent en charge des patients indemnes et des porteurs.
- Dépister les contacts toutes les semaines tant que l'épidémie n'est pas contrôlée et qu'un porteur est présent
- Renforcer l'accompagnement par l'EOH des équipes du service, de jour et de nuit, y compris les fins de semaine, pour assurer un haut niveau de respect des précautions standard, notamment l'hygiène des mains.
- Renforcer le bionettoyage de l'environnement et du matériel partagé, (ex pèse-personnes, échographe, zone de préparation des soins, zones fréquemment touchées comme les claviers d'ordinateurs, …)
- Prélèvements d'environnement selon avis de l'EOH.
- Mettre en place un dispositif de repérage informatique et d'alerte lors des ré-hospitalisations des cas et des contacts.
- En cas de ré-hospitalisation, placer les patients contact en PCC et les dépister.
- Epidémie considérée comme contrôlée après 3 dépistages négatifs des contacts hors exposition.

Lorsque l'épidémie est contrôlée, après la sortie des porteurs, il est possible d'arrêter les dépistages et de retirer des listes de suivi les patients contact à risque élevé dont au moins 3 dépistages successifs réalisés à une semaine d'intervalle et hors exposition sont négatifs. Après un an sans nouveau cas, retirer les contacts non dépistés des listes de suivi.

SIGNALEMENT ET ENVOI DES SOUCHES AU CNR MYCOSES INVASIVES ET ANTIFONGIQUES (CNRMA)

Tout cas confirmé (infecté ou colonisé) doit faire l'objet d'un signalement à l'EOH de l'établissement, à celle de l'établissement d'accueil en cas de transfert, à l'EOH du siège et sur e-SIN.

Les souches doivent être envoyées au CNRMA (Pasteur) pour confirmation d'identification et analyse génotypique.

Rédaction : Clarisse Duverger, Sandra Fournier, Valérie Souyri
Relecture : Alexandre Alanio, Christine Bonnal, Jean-Winoc Decousser, Pierre Frange, Maud Gits-Muselli, Christophe Hennequin, Najiby Kassis-Chikhany, Jean-Christophe Lucet, et les équipes opérationnelles d'hygiène de l'AP-HP

Références :

- ECDC Rapid risk assessment 17/02/2022. Candida auris outbreak in healthcare in Northern Italy, 2019-2021
-HCSP, « Mesures de prise en charge de patient infecté ou colonisé par Candida auris », Haut Conseil de la Santé Publique, Paris, juin 2019. Consulté le: 8 février 2022. [En ligne]. Disponible sur: https://www.hcsp.fr/explore.cgi/avisrapportsdomaine?clefr=730
- Proctor DM et al. Integrated genomic, epidemiologic investigation of Candida auris skin colonization in a skilled nursing facility Nat Med. 2021 Aug;27(8):1401-1409.
-Infection Prevention and Control for Candida auris | Candida auris | Fungal Diseases | CDC, 12 juillet 2021. https://www.cdc.gov/fungal/candida-auris/c-auris-infection-control.html (consulté le 8 février 2022).
-Antimicrobial products registered with EPA for claims against Candida auris, US environmental protection angency, Lien
-D. W. Eyre et al., « A Candida auris Outbreak and Its Control in an Intensive Care Setting », N Engl J Med, vol. 379, no 14, p. 1322-1331, oct. 2018, doi: 10.1056/NEJMoa1714373.
-D. Plachouras, F. Lötsch, A. Kohlenberg, D. L. Monnet, et the C. auris survey collaborative Group, « Candida auris: epidemiological situation, laboratory capacity and preparedness in the European Union and European Economic Area*, January 2018 to May 2019 », Eurosurveillance, vol. 25, no 12, p. 2000240, mars 2020, doi: 10.2807/1560-7917.ES.2020.25.12.2000240.
-J. Rhodes et M. C. Fisher, « Global epidemiology of emerging Candida auris », Curr Opin Microbiol, vol. 52, p. 84-89, déc. 2019, doi: 10.1016/j.mib.2019.05.008.
-S. Tsay, A. Kallen, B. R. Jackson, T. M. Chiller, et S. Vallabhaneni, « Approach to the Investigation and Management of Patients With Candida auris, an Emerging Multidrug-Resistant Yeast », Clin Infect Dis, vol. 66, no 2, p. 306-311, janv. 2018, doi: 10.1093/cid/cix744.
-G Desoubeaux, AT Coste, C Imbert, C Hennequin. Overview about *Candida auris*: What's up 12 years after its first description? Journal of Medical Mycology 32(2022).

Table S1: E-test and EUCAST minimal inhibitory concentrations performed in isolates from P0 and P1

Patient	Strain ID	Specimen	Date of sampling	Method	AMB	5-FC	FLC	VRC	PSC	CSP	MCF
P0	na	Blood culture	29/01/2021	Etest	0.5	0.064	> 256.0	0.125	nd	nd	0.064
P0	CNRMA21-088	Burn wound (thigh)	02/02/2021	EUCAST	0.25	≤0.125	32	0.125	≤0.016	0.015	0.015
P0	CNRMA21-086	BAL	02/06/2021	EUCAST	0.25	≤0.125	32	0.125	≤0.016	0.03	0.015
P0	CNRMA21-087	Catheter	02/07/2021	EUCAST	0.25	≤0.125	32	0.125	≤0.016	0.015	0.015
P0	na	Burn wound (arm)	05/02/2021	Etest	0.75	0.064	> 256.0	0.125	nd	nd	0.064
P0	na	Burn wound (abdomen)	10/02/2021	Etest	0.38	0.064	> 256.0	0.064	nd	nd	0.094
P1	CNRMA21-252	Burn wound (thigh)	03/04/2021	EUCAST	0.25	≤0.125	32	0.25	≤0.016	≤0.008	0.015

AMB, amphotericin B; 5-FC, flucytosine; FLC, fluconazole; VRC, voriconazole ; PSC, posaconazole ; CSP, caspofungin ; MCF, micafungine, nd, not done ; na, non available
